# Clinical, haematological and biochemical findings in tigers infected by *Leishmania infantum*

**DOI:** 10.1186/s12917-020-02419-y

**Published:** 2020-06-22

**Authors:** Maria Alfonsa Cavalera, Roberta Iatta, Pietro Laricchiuta, Giuseppe Passantino, Francesca Abramo, Jairo Alfonso Mendoza-Roldan, Domenico Otranto, Andrea Zatelli

**Affiliations:** 1 Department of Veterinary Medicine, University of Bari, 70010 Valenzano, Italy; 2 Zoo Safari di Fasano, Fasano, 72015 Brindisi, Italy; 3 Department of Veterinary Science, University of Pisa, Pisa, 56124 Pisa, Italy; 4 Faculty of Veterinary Sciences, Bu-Ali Sina University, Hamedan, Iran

**Keywords:** Tiger, Leishmaniasis, Symptomatology, Laboratory abnormalities, Dysproteinemia

## Abstract

**Background:**

A large number of animal species are susceptible to *Leishmania infantum* (Kinetoplastida, Trypanosomatidae) in endemic areas, including domestic and wild felids such as tigers (*Panthera tigris*). Knowledge on the infection of this endangered species is still at its infancy, and therefore this study aims to identify clinical presentation and clinicopathological findings of tigers naturally infected by *L. infantum*.

**Results:**

Tigers either *L. infantum*-positive (group A) or -negative (group B) were apparently healthy or presented visceral leishmaniasis unrelated conditions, except for one animal in which a large non-healing cutaneous lesion was observed. However, histological exam and immunohistochemistry carried out on the lesion excluded the presence of *L. infantum* amastigotes. Biochemical analysis showed that the average concentration of total proteins, globulins and haptoglobin were significantly higher (*p* < 0.01, *p* = 0.01 and *p* = 0.02, respectively), while the albumin/globulin ratio significantly lower (*p* = 0.05) in group A compared with group B. The biochemical alterations were partially confirmed by the serum protein electrophoresis results revealing a significant increase in the total protein value (*p* = 0.01) and hypergammaglobulinemia (*p* = 0.03) but an unmodified albumin/globulin ratio in group A.

**Conclusions:**

In this study tigers infected by *L. infantum* have shown to be mainly asymptomatic. The absence of clinical signs may lead veterinarians to overlook leishmaniasis in animals kept in captivity. Therefore, diagnostic and screening tests as serology should be part of routinely surveillance programs to be performed on tigers in zoological gardens located in endemic areas. Though only few protein-related laboratory abnormalities were recorded in infected animals, they could provide diagnostic clues for a first suspicion of *L. infantum* infection in tigers. Indeed, considering the high risk of zoonotic transmission in heavily frequented environment as zoos, a prompt diagnosis of *L. infantum* infection is of pivotal importance.

## Background

*Leishmania infantum* (Kinetoplastida, Trypanosomatidae) has been recognised as the main causative agent of canine leishmaniasis (CanL) [1]. This zoonotic sand fly-borne disease is one of the world’s most important neglected illnesses [2], though it is potentially fatal to humans if left untreated [3]. Dogs are the main peridomestic reservoir host of the protozoa [4], developing a chronic and multisystemic infection that may potentially involve any organ [5]. Consequently, CanL is characterized by a wide variety of non-specific clinical presentations ranging from asymptomatic infection to a severe fatal clinical disease, mainly depending on the host’s Th1/Th2 immune responsiveness [6, 7]. The most common clinical manifestations of CanL due to *L. infantum* are skin lesions, lymphadenomegaly and weight loss [7, 8].

In endemic areas, *L. infantum* is maintained by several mammal species in nature [9] and the role of wildlife species (e.g., red fox, golden jackal, gray wolf, hare, black rat) has been suggested in a series of studies [10, 11]. After being long regarded as resistant, cats have gain the interest of the scientific community as hosts of *L. infantum*, mainly in endemic areas [12, 13]. Clinical signs compatible with feline leishmaniasis (FeL) include lymphadenomegaly, splenomegaly, weight loss, anorexia, as well as cutaneous, mucocutaneous and ocular lesions [14]. However, the subclinical infection seems more common in endemic regions such as Mediterranean countries [14]. The complex pathogenesis and the broad spectrum of clinical manifestations of visceral leishmaniasis (VL) in domestic animals make its diagnosis still challenging [15]. Although the diagnosis of VL can be achieved by performing specific tests (i.e., serology, cytology, PCR, xenodiagnosis), laboratory abnormalities uncovered by routine haematology, clinical chemistry, or urinalysis may support the clinical suspicion of VL [15]. For example, in CanL, nonregenerative normocytic normochromic anemia, leukogram changes (i.e. neutrophilia), trombocytopenia and dysproteinemia (i.e., hyperproteinemia, hyperglobulinemia, hypoalbuminemia, inversion in the albumin/globulin ratio) represent typical laboratory findings [1, 15, 16]. Renal dysfunction may be revealed by clinical chemistry (renal azotemia) and urinalysis (decreased urine specific gravity and proteinuria). Serum protein electrophoresis (SPE) is commonly used to identify a polyclonal gammopathy that may also appear oligo-monoclonal, especially in dogs coinfected by other vector-borne pathogens [1, 15]. Additionally, the increase of acute phase proteins (APP) and other markers of inflammation (i.e., C-reactive protein [CRP], serum amyloid A [SAA], haptoglobin [HPT], ferritin) have been frequently associated with CanL [15, 16].

In cats, scant information is available about laboratory abnormalities in *L. infantum* infected individuals as only the major body of literature is represented by case reports. Mild to severe normocytic normochromic non-regenerative anemia and hyperproteinemia with hypergammaglobulinemia are the main abnormalities reported [17, 18] followed by moderate to severe pancytopenia in association with aplastic bone marrow, particularly in FIV-positive cats. Unlike in dogs, hypoalbuminemia is only occasionally detected in FeL [17]. Recently, wild species of felids, including tigers (*Panthera tigris*), have been recognized as susceptible hosts to *L. infantum* in endemic areas [19, 20]. Considering that knowledge of the infection of this endangered species [21] is still at its infancy, data on clinical picture in tigers infected by *L. infantum* are missing. Thus, the aim of this study was to identify clinical presentation and clinicopathological findings of tigers naturally infected with *L. infantum*.

## Results

### Clinical findings

Tigers (8 males and 12 females), individually identified by microchip, aged from 6 months to 11 years, weighed from 70 to 220 kg and showed a BCS = 3 except for the tiger 4 (BCS = 2) (Table 1). Animals from group A (i.e. *L. infantum*-positive animals) as well as those from group B (i.e *L. infantum*-negative animals) were apparently healthy or presented VL unrelated conditions, except for the tiger 1 in which a large (10 cm) non-healing cutaneous lesion was observed. After an incisional skin biopsy of the lesion, an histological exam has been performed revealing a chronic ulcerative dermatitis and vasculitis/perivasculitis with plasma cellular infiltrate. During the histological examination, no pathogens were detected. The absence of amastigotes was also confirmed by immunohistochemistry.

**Table 1:** Data on sex, age, body weight, body condition score (BCS), serological and molecular results for *Leishmania infantum* of the 9 and 11 tigers enrolled in group A (*L. infantum*-positive animals) and group B (*L. infantum*-negative animals)

Group	Tiger ID	Sex (M/F)	Age (years)	Weight (kg)	BCS (1–5)	IFAT/Ab titre	qPCR results	
							Lymph node	Swab (conjuntival, oral, nasal)
A	1	F	7	150	3	1:160	pos	neg
	2	M	6	160	3	1:80	nt	neg
	3	M	7	220	3	1:80	neg	neg
	4	M	8	210	2	1:80	nt	pos
	7	F	2	135	3	1:640	pos	pos
	9	F	2	150	3	1:40	pos	neg
	10	M	2	170	3	1:40	pos	pos
	11	F	8	190	3	1:40	neg	neg
	17	F	7	150	3	1:80	neg	neg
B	5	M	7	220	3	neg	neg	neg
	6	F	9	150	3	neg	neg	neg
	8	M	2	180	3	neg	nt	neg
	12	M	6	220	3	neg	neg	neg
	13	F	7	160	3	neg	neg	neg
	14	F	11	120	3	neg	neg	neg
	15	M	1	130	3	neg	neg	neg
	16	F	1	110	3	neg	neg	neg
	18	F	0.5	70	3	neg	neg	neg
	19	F	0.5	70	3	neg	neg	neg
	20	F	0.5	70	3	neg	neg	neg

*nt* not tested

### Haematological and biochemical findings

Mean and median values of complete blood count (CBC) and biochemical parameters obtained from group A and B are statistically compared and reported in Tables 2 and 3, respectively. The SPE results are shown in Table 3. Data available on the Zoological Information Management System (ZIMS) from Species 360 (formerly the International Species Inventory System, ISIS) have been included, when available, as haematology and biochemistry reference values of the tigers (Tables 2, 3). Biochemical analysis showed that the average concentration of total proteins, globulins and haptoglobin were significantly higher (*p* < 0.001, *p* = 0.01 and *p* = 0.02, respectively), while the albumin/globulin ratio significantly lower (*p* = 0.05) in group A compared with group B. Urine samples were collected from 12 (i.e. *n* = 6 *L. infantum*-positive tigers and *n* = 6 *L. infantum*-negative tigers) out of 20 animals. Mean and median values of urine specific gravity (USG) and urine protein/creatinine ratio (UP/C) detected in group A and B are reported in Table 3.

**Table 2:** Mean and median (in brackets) values of complete blood count (CBC) parameters obtained from group A (*L. infantum*-positive tigers) and B (*L. infantum*-negative tigers)

CBC parameters	Reference values (ZIMS)	Group A (*n* = 9)	Group B (*n* = 11)	*p* value
RBC (*× 10*^6^*/*μL)	6.98 (7.01)	7.5 (7.7)	7.3 (7.3)	0.49
HGB (g/dL)	13.3 (13.5)	13 (13)	13.4 (13.5)	0.64
HCT (%)	40.1 (40.2)	39.3 (40.1)	39.8 (39.8)	0.83
PLT	238 (229)	322.6 (322)	298.3 (297)	0.19
WBC (× 10^3^/μL)	10.4 (10)	14.6 (13.7)	12.9 (13.2)	0.42
Band neutrophils (/μL)		58.1 (0)	82.5 (0)	0.88
Segmented neutrophils (/μL)	7490 (7330)	12,214.2 (11371)	10,859.2 (10,693.5)^a^	0.50
Lymphocytes (/μL)	1450 (1340)	1549.4 (1344)	1440.7 (1610)^a^	0.69
Monocytes (/μL)	301 (260)	350.4 (322)	335.4 (312)^a^	0.60
Eosinophils (/μL)	191 (160)	427.8 (350)	166 (167)^a^	0.09

Significant *p* values are printed in bold

ZIMS Species 360 (Zoological Information Management System 2017)

^a^Parameter obtained from 10 out of 11 *L. infantum*-negative tigers

**Table 3:** Mean and median (in brackets) values of biochemical, serum protein electrophoresis and urinalysis values obtained from group A (*L. infantum*-positive tigers) and B (*L. infantum*-negative tigers)

Parameters	Reference values (ZIMS)	Group A (*n* = 9)	Group B (*n* = 11)	*p* value
Biochemical profile				
CK (IU/L)	242 (199)	258.6 (200)	160.2 (153)	0.29
AST (IU/L)	31 (26)	27.7 (23)	25 (22)	0.37
ALT (IU/L)	73 (62)	47.2 (43)	46.1 (47)	0.66
ALP (IU/L)	27 (20)	19.3 (13)	114.3 (40)	0.07
GGT (IU/L)	2 (1)	0.5 (0.7)	0.7 (0.7)	0.52
Total bilirubin (mg/dL)	0.2 (0.1)	0.2 (0.2)	0.1 (0.1)	0.08
Glucose (mg/dL)	144 (135)	119.1 (125)	121.6 (124)	0.86
BUN (mg/dL)	31.4 (30)	64.3 (63)	60.9 (61)	0.42
Creatinine (mg/dL)	2.5 (2.5)	2.5 (2.5)	2.2 (2.4)	0.41
Serum iron (μg/dL)	99 (92)	68.6 (68)	80 (83)	0.22
TIBC (μg/dL)	306 (293)	251.1 (243.5)	285.5 (280)	**0.04**
UIBC (μg/dL)		181.7 (185)	205.5 (219)	0.13
Total proteins (g/dL)	7.1 (7.1)	8.7 (8.7)	7.5 (7.3)	**0.003**
Albumin (g/dL)	3.7 (3.7)	3.2 (3.3)	3.3 (3.4)	0.40
Globulins (g/dL)	3.5 (3.4)	5.5 (5.9)	4.1 (4.2)	**0.01**
Albumin/Globulins ratio (g/dL)	1.1 (1.1)	0.6 (0.5)	0.8 (0.8)	**0.05**
HPT (mg/dL)		155.5 (171.9)	92.5 (85.2)	**0.02**
CRP (mg/dL)		7.1 (5.9)	9.3 (8.5)	0.22
SAA (μg/mL)		145.3 (24.4)	42.8 (1.2)	0.20
Serum protein electrophoresis				
Total proteins (g/dL)	7.1 (7.1)	8.7 (8.7)	7.6 (7.4)	**0.01**
Albumin (g/dL)	3.66 (3.7)	3.5 (3.5)	3.7 (3.7)	0.34
α-1 globulins (g/dL)	0.38 (0.3)	0.2 (0.3)	0.3 (0.3)	0.09
α-2 globulins (g/dL)	0.53 (0.47)	1.0 (1.0)	0.7 (0.7)	0.07
β globulins (g/dL)	0.81 (0.72)	1.0 (1.0)	1.0 (1.0)	0.72
γ globulins (g/dL)	1.57 (1.61)	2.9 (3.2)	1.9 (1.9)	**0.03**
Albumin/Globulins ratio		0.8 (0.6)	1.1 (0.9)	0.08
Urinanalysis				
PS	1034 (1035)	1056.8 (1057)^a^	1054 (1055.5)^b^	0.68
UP/C	0 (0)	0.2 (0.2)^a^	0.2 (0.1)^b^	0.31

Significant *p* values are printed in bold

ZIMS Species 360 (Zoological Information Management System 2017)

^a^Parameter obtained from 6 out of 9 *L. infantum*-positive tigers

^b^Parameter obtained from 6 out of 11 *L. infantum*-negative tigers

## Discussion

This study represents the first identification of clinical presentation and clinicopathological findings in *L. infantum* naturally infected tigers. These animals have shown to be asymptomatic considering the clinical pictures usually detected in CanL and FeL [7, 8, 14]. Although data on the pathogenesis of *L. infantum* infection in tigers are missing, it can be supposed that progression to illness could be associated with increasing antibody titres as it occurs in CanL and, likely, in FeL [6, 14]. Indeed, out of 8 asymptomatic tigers positive at IFAT, 7 presented a low antibody titre (i.e., *n* = 3 1:40, *n* = 4 1:80).

In this study, no statistically significant haematological differences were found between tigers of group A and B (Table 2). Similarly, haematological and biochemical parameters are usually not modified in *L. infantum* infected dogs with or without mild localized clinical signs but positive to direct diagnostic methods or presenting low-titre or none anti-*Leishmania* antibodies [7, 16, 22].

Conversely, a significant protein imbalance including hyperproteinemia, hyperglobulinemia and decreased albumin/globulin ratio were detected in group A (Table 3). These abnormalities in the biochemistry panel can appear early during the onset of VL [15] being associated with disease progression [16] and represent a typical finding in CanL, as also described in cases of cats with FeL [15, 17, 18]. In addition, no difference in the levels of albumin in the group A compared to group B was found, similarly to cats where hypoalbuminemia has been only occasionally detected [17].

The biochemical alterations were partially confirmed by the SPE results revealing a significant increase in the total protein value (*p* = 0.01) and a hypergammaglobulinemia (*p* = 0.03) but an unmodified albumin/globulin ratio in group A (Table 3). Furthermore, even though a significant increase of α-2 globulins in group A (1.0 g/dL) compared to group B (0.7 g/dL) was not found, the value of this globulin in *L. infantum*-positive animals was moderately increased referred to the ZIMS reference parameter for tigers (0.53 g/dL) (Table 3). α-2 globulins includes most of the positive APP, as haptoglobin, which are powerful indicators of inflammation during CanL [15]. Therefore, the lightly increased of α-2 globulins in group A can be explained by the significantly increase of the haptoglobin (*p* = 0.02) detected in *L. infantum*-positive tigers (Table 3).

The presence of renal disease in group A can be excluded considering the absence of alteration in renal function markers as the serum concentration of creatinine and BUN, the urine specific gravity and the urine protein/creatinine ratio (Table 3). These markers are useful to suspect severe/very severe CanL with renal involvement [16] causing a chronic kidney disease (CKD) that is a well-recognized condition potentially fatal for dogs affected by CanL [15]. Though renal failure in association with FeL has been described, the exact role of *L. infantum* infection in the development of multiorgan injury with renal impairment has to be confirmed [17]. Therefore, considering the lack of information on leishmaniasis in tigers, further studies are necessary to draw any conclusion on the potential non-involvement of the kidney in this new species.

The clinicopathological picture of *L. infantum-*infected tigers emerging from the present study is mainly characterized by a subclinical condition and few protein-related laboratory abnormalities, which highly supports the hypothesis on the potential role of tigers as alternative local reservoir of *L. infantum*. Similarly, this picture has been suggested also for cats, which presumably act as reservoir hosts at least in endemic areas of zoonotic VL [12–14].

Although laboratory abnormalities are usually aspecific in animals with VL [15], they can represent a wake-up call for veterinarians working with tigers in zoological parks where routine screening for VBDs as leishmaniasis is not frequent. Furthermore, performing laboratory tests could be essential in *L. infantum*-positive tigers which live in an endemic area, thus being at risk of infection (*n* = 9/20, 45%). However, the potential limitation of the study consists of the impossibility of carrying out an in-depth clinical examination that would allow all clinical signs related to VL (e.g., splenomegaly) to be properly evaluated. Another limit of the study could be represented by the absence of information on potential other diseases affecting tigers which can influence the laboratory parameters. Finally, the available haematological and biochemical reference values in tigers are limited and obtained from a small population size not properly divided considering age, sex and sampling season [23].

## Conclusions

In this study tigers infected with *L. infantum* have shown to be mainly asymptomatic considering the clinical pictures classically observed in CanL and FeL. The complete absence of clinical signs may lead veterinarians to overlook leishmaniasis in animals kept in captivity where the health condition of the animals is usually checked by periodical visual examinations. Therefore, diagnostic and screening test as serology should be part of routinely surveillance programs to be performed on tigers in zoological gardens located in endemic areas. Though only few protein-related laboratory abnormalities have been detected in infected animals, they can provide essential diagnostic clues for identification of *L. infantum* infection in tigers. This may play an important role in the conservation of this endangered species of wild felids as well as in reducing the risk of zoonotic transmission in heavily frequented environment as zoos.

## Methods

### Study area and animals’ enrolment

Twenty tigers housed at a Safari Park (Apulia region, Brindisi Province, southern Italy) (40°50′ N, 17°20′ E) were involved in the study. Those animals have been previously enrolled in an epidemiological investigation aimed at evaluate the prevalence of *L. infantum* infection after the identification of an index case in the tiger population, using both serological and molecular methods. Nine (45%) out of 20 tigers were positive to *L. infantum* by immunofluorescence antibody test (IFAT) (9/20, 45%) and/or by real time-PCR (5/20, 25%), being five of them (5/20, 25%) positive by both methods.

Out of 9 serologically positive tigers, three animals presented an antibody titre of 1:40, four of 1:80, one of 1:160 and one of 1:640 each, respectively. Therefore, according to these results [24] in the overall tiger population two groups were set up: *L. infantum*-positive animals (*n* = 9; group A) and *L. infantum*-negative animals (*n* = 11; group B).

### Clinical evaluation

Food and water were withheld for 12 h prior to anaesthesia. Upon arrival at the zoological park, each animal was anesthetised, via remote injection, with a combination of 250 mg of zolazepam/tiletamine (Zoletil 50/50, Virbac) plus 3 mg of detomidine (Mesden 10 mg/ml, FM Italia) [25]. Then, a complete physical exam was performed, and for each animal body condition score was assessed [26]. The clinical visit included a whole-body visual examination for cutaneous lesions and lymph nodes evaluation. After verifying sex and reproductive status, external genitalia and perineum were inspected. Mucous membrane colour and moisture were observed. Other visual observations included evaluation for normal breathing patterns and rate.

At the end of the procedures (i.e. 30–45 min after darting), immobilization of the tigers was reversed with intramuscular administration of 15 mg of atipamezole (Antisedan 0.5%, Pfizer, Milan, Italy) [25]. After recovery (i.e. ≃10 min), each tiger was closely monitored for 8 h and, thereafter, reintroduced into the colony of the zoo.

### Blood samples and analysis

Blood (12 mL) was collected from the cephalic vein and placed in tubes with EDTA (2 mL) to perform routine haematology, and in plain tubes (10 mL) to obtain serum. Serum was obtained within 4 h from collection, by centrifugation (15 min at 1500 x g).

Results from complete blood count (CBC) (Siemens, ADVIA 2120), serum biochemical analysis (Beckman Coulter, Clinical Chemistry Analyzer AU680) and electrophoresis (SEBIA, Capillarys 2 Flex Piercing) were achieved with the same methods in all samples.

### Urine collection and urinalysis

All urine samples were collected by catheterization, using a 20 mL syringe connected to a 14 Ch catheter. Urine samples were placed in 10 mL, sterile, evacuated collection tubes, stored at room temperature (approximately 20 °C [676 °F]), and analysed within 4 h from collection. Urine sediment was obtained by centrifugation (10 min at 900 x g) of 5 mL of urine, followed by removal of 4.5 mL of supernatant, and resuspension of the remaining 0.5 mL of urine. A sample of 12 μL of the resuspended urine was microscopically assessed. Red blood cells and white blood cells were expressed as mean number of cells/10 hpf (40 × magnification). Urine sediment with bacteriuria, and/or > 5 red blood cells or white blood cells/hpf, was considered indicative of active inflammation and excluded from the UPC ratio evaluation. The supernatant was transferred into separate tubes to determine UPC ratio. To calculate the UPC ratio, protein concentration (mg/dL) was measured with pyrogallol red, and creatinine (mg/dL) was measured using the Jaffé method on undiluted urine supernatant that was thawed before analysis. Results were achieved with the same method in all samples (Beckman Coulter, Clinical Chemistry Analyzer AU680).

### Data analysis

The Kolmogorov-Smirnov test was used to assess the normality of the data. Statistical differences between group A and group B were tested for significance by Student’s t-test for normally distributed data and with the Mann-Whitney test for not normally distributed data. A value of *P* < 0.05 was considered statistically significant. The statistical analyses were performed using GraphPad Prism version 8.0.0 (GraphPad Software, San Diego California, USA).

## Data Availability

Data sets generated from this study are available upon request to the corresponding author.

## Abbreviations

ALP: Alkaline phosphatase
ALT: Alanine aminotransferase
AST: Aspartate aminotransferase
BUN: Blood urea nitrogen
CanL: Canine leishmaniasis
CBC: Complete blood count
CK: Creatine kinase
CRP: C-reactive protein
EDTA: Ethylenediamine tetraacetic acid
FeL: Feline leishmaniasis
GGT: Gamma glutamyltransferase
HCT: Hematocrit
HGB: Hemoglobin
HPT: Haptoglobin
IFAT: Immunofluorescence antibody test
PLT: Platelet
RBC: Red blood cell
SAA: Serum amyloid A
SPE: Serum protein electrophoresis
TIBC: Total Iron Binding Capacity
UIBC: Unsaturated iron binding capacity
UP/C: Urine protein creatinine ratio
VL: Visceral leishmaniasis
WBC: White blood cell
